# Relation between microRNAs and Apoptosis in Hepatocellular Carcinoma

**DOI:** 10.3889/oamjms.2016.038

**Published:** 2016-03-09

**Authors:** Refaat R. Kamel, Khalda Said Amr, Mie Afify, Yasser A. Elhosary, Abdelfattah E. Hegazy, Hoda H. Fahim, Wafaa M. Ezzat

**Affiliations:** 1 *Surgery Department, Faculty of Medicine, Ain Shams University, Cairo, Egypt*; 2 *Medical Molecular Genetics Department, National Research Center, Cairo, Egypt*; 3 *Biochemistry Department, National Research Centre, Cairo, Egypt*; 4 *Internal Medicine Department, National Research Centre, Cairo, Egypt*; 5 *Surgery Department, Elsahel Teaching Hospital, Cairo, Egypt*; 6 *Anesthesia Department, Elsahel Teaching Hospital, Cairo, Egypt*

**Keywords:** HCC, apoptosis, pathogenesis, microRNAs, HCV

## Abstract

**AIM:**

To determine the relation between serum microRNAs and apoptotic markers as regards development of HCC to understand the underlying mechanism of HCV related hepatocarcinogenesis.

**PATIENTS AND METHODS:**

A total of 65 serum samples (25 samples from controls, 20 samples from hepatitis and 20 samples from HCC patients) were collected for miRNAs (mir 21, mir 199-a, and mir 155) detection. Human Programmed cell death protein-4 (PDCD-4) and Human Cytochrome-C (CYT-C) were determined.

**RESULTS:**

miRNAs 21 and 155 were over expressed in sera of patients with HCC compared to patients with chronic hepatitis (p < 0.0001). While serum means values of miR 199a was significantly decreased among HCC group patients when compared to patients with chronic hepatitis (p < 0.0001). The serum levels of PCDC4 and CYTC were increased in patients with HCC when compared to chronic hepatitis patients. They were also increased in patients with chronic hepatitis when compared to controls (p < 0.05, significant). There was direct correlations between apoptotic markers and oncomirs miRNAs 21 and 155 while apoptotic markers were inversely correlated with miRNA 199-a.

**CONCLUSION:**

Both microRNAs and apoptotic markers have roles in HCC pathogenesis. It seems that oncogenic microRNAs induce liver carcinogenesis in HCV patients irrespective of suppression of apoptosis.

## Introduction

Liver cancer is one of the most common malignancies all over the world and among the important causes of malignancy-related death [1]. Similar to other malignancies, the pathogenesis of liver cancer is a complex with contribution of genetic and epigenetic changes [2].

MicroRNAs (miRNAs) are a class of phylogenetically conserved short RNAs that suppress protein expression through base-pairing with the 3’-untranslated region (3’-UTR) of target mRNA [3]. Many studies suggest that miRNAs act significant roles in diverse biological processes and the dysfunction of miRNAs is included in the cancer development [4].

Cell cycle dysregulation is an important step in the induction and development of human malignancies, including liver cancer. Accumulating evidence has shown that deregulated miRNAs may affect HCC cell proliferation through direct interaction with critical regulators of cell cycle machinery [5].

Apoptosis is a natural barrier to tumorigenesis and malinancy progression. Cancer cells struggle to avoid apoptosis to escape from the supervision of the body and to survive in the difficult tumor environment [6].

There are many miRNAs that target anti-apoptotic members of the Bcl-2 family. Most are significantly downregulated in HCC. For instance, miR-16 and miR-29 are down regulated in HepG2 cells, and one of their target genes is confirmed to be Bcl-2 [7]. There are some other miRNAs whose target gene is Mcl-1. Apart from silencing of Bcl-2, miR-29 can also directly target Mcl-1 in mitochondrion-mediated apoptotic pathway [8]. In addition, miR-101, miR-193b, miR-125b, and let-7c, which are downregulated in HCC cells, might exert anti-apoptotic action via targeting Mcl-1 [9].

Xiong and his colleagues have reported a significant down-regulation of miR-29 family members, including miR-29a, miR-29b, and miR-29c (miR-29a/b/c), in tissues of liver cancer [10]. This is in accordance with previous observations in other types of human neoplasm [11]. It has been shown that ectopic expression of miR-29b inhibits cell growth and promotes tumor necrosis factor–related apoptosis inducing ligand–triggered apoptosis [12].

In this study, we found differentially deregulated microRNAs (mir21; mir199-a; mir155) among patients with HCV related hepatocellular carcinoma. Moreover, apoptotic markers: Programmed Cell Death Protein 4 (PDCD4) and Cytochrome C (CYTC) were up regulated in the same patients. Here we try to clarify the relation between serum microRNAs and apoptotic markers as regards development of HCC to understand the underlying mechanism of HCV related hepatocarcinogenesis.

## Patients and Methods

Between June 2011 and June 2013, a total of 65serum samples (25 samples from controls, 20 samples from hepatitis and 20 samples from HCC patients) were collected from patients who underwent liver resection or living donor liver transplantation (LDLT) at the First Affiliated El Sahahel Teaching Hospital and Dr. Refaat Kamel Hospital (Cairo, Egypt). Serum samples were also collected from 25 healthy volunteers who served as control group. All of the HCC patients were diagnosed by liver biopsy or by the findings of at least two radiological tests of HCC, including abdominal ultrasound, magnetic resonance imaging (MRI), hepatic angiography and contrast-enhanced dynamic computed tomography or by increased AFP (AFP ≥200 μg/mL). Patients with secondary or recurrent tumors, a history of other malignant tumors; patients with hepatitis B virus infection or being included in other studies were excluded from this study. For the 20 chronic hepatitis cases, the diagnosis was based on the serum tests. Serum hepatitis B surface antigens (HBsAg) and anti-HCV antibody were assayed by microparticle enzyme immunoassay using commercial kits to determine hepatitis B or hepatitis C infection. A total of 25 cancer-free controls were attached at the physical examination center. Controls that had clinical liver diseases were excluded. The current study was approved by Ethical Committee of National Research Center. After signing an informed consent, all subjects were asked to fill a questionnaire to investigate the demographic characteristics, disease history, and the history of cancer and alcohol or tobacco use. Consent to publish data of the current research was obtained from every participant. The clinical characteristics including tumor differentiation, tumor size, metastasis, Child-Pugh class, were collected from medical records.

### miRNA extraction and quantification

Isolation of miRNAs from blood of patients and controls followed the protocol for miRNeasy RNA isolation kit (Qiagen, Germany). Separation of serum took place immediately within 2 h from blood sample collection. The extracted total RNA including miRNA from tissue and serum are subjected to reverse transcription.

### RT-PCR

TaqMan miRNA assays (Life Technologies, CA) were used to quantify the expression levels of mature miR-122 Total RNA extracted by miRvana (life technologies) was reverse transcribed in reaction mixture containing miR-specific stem-loop RT primers. Quantitative real time polymerase chain reaction (qPCR) was performed with 3 microliter of each cDNA on aStep One TM Plus Real-Time PCR System (ABI) in duplicates reactions containing the prepared cDNA and TaqMan specific primers in Universal Master Mix without Amp Erase UNG (Applied Biosystems) and threshold cycles (CT) were calculated using Sequence Detection Software (SDS v2.2.1, Applied Biosystem). All mRNA quantification data were normalized to 18S RNA. All miRNA data are expressed relative to a RNU48 small nuclear (sn)RNA TaqMan PCR performed on the same samples. Fold expression was calculated from the mean CT values using the 22DDCt method. Relative quantity (RQ) of miRNAs 21 and 199-a was calculated by the formula (RQ=2−ΔΔCt), where Ct is defined as the fractional cycle number at which the fluorescence generated by cleavage of the probe passes a fixed threshold above baseline.

### Detection of Human Programmed cell death protein-4 (PDCD-4) and Human Cytochrome-C (CYT-C) in blood samples (serum)

Detection of apoptotic markers in serum was carried out using this assay employed the quantitative sandwich enzyme immunoassay technique. The kits (Glory Science Co., Ltd, USA) use double – antibody sandwich enzyme- linked immunosorbent assay (ELISA) to assay the level of Human Programmed cell death protein 4 (PDCD4) and Human Cytochrome C (CYTC) in blood samples (serum). The procedure for each parameter is as follows:

◾ Add PDCD4 to monoclonal antibody Enzyme well which is pre-coated with Human Programmed cell death protein monoclonal antibody, incubation;, or add CYTC to monoclonal antibody Enzyme well which is pre-coated with Human cytochrome C monoclonal antibody; then
◾ Add PDCD4 or Cytochrome c (CYTC) antibodies labeled with biotin, and combined with Streptavidin-HRP to form immune complex; then carry out incubation and washing again to remove the uncombined enzyme. Then add Chromogen Solution A, B, the color of the liquid changes into the blue, and the effect of acid, the color finally becomes yellow. The chroma of color and the concentrations of the human substance Programmed cell death 4 (PDCD4) or Cytochrome C (CYTC) of sample were positively correlated.
◾ We calculate the O.D value of all the wells with the standard and the wells with samples, make the standard curve diagram with concentration of the standard from the low to the high and the left to the right as abscissa {X} and O.D values of the wells at 450nm as ordinate {Y} axis.
◾ Then find out the corresponding concentration range of each sample on this standard curve diagram according to their O.D values.

### Statistical analysis

Data are expressed as mean ± SD unless otherwise indicated. Categorical data are described as frequency of the subjects with a specific characteristic. Chi-square test or Fisher’s exact test was used for comparing categorical data and Student’s t-test, Mann-Whitney-U-test, one-way ANOVA or Kruskal-Wallis test, when appropriate, was used for comparing continuous variables. Two-tailed p-values less than 0.05 were considered statistically significant. Statistical analysis was performed using SPSS software version 12.0 (SPSS Inc., Chicago, IL, USA).

## Results

### General characteristics for the studied patients

Two groups of chronic HCV patients were enrolled in the current study; HCC group consists of 20 patients, 19 males and one female with mean age of 56.25 ± 8.13 years. Chronic hepatitis group consists of 20 patients, 15 males and 5 females with mean age of 46.22 ± 8.45years. Frequency of past history of Schistosomiasis was 55% among HCC group while it was 60% among patients of chronic hepatitis group as shown in Table 1. There were no any significant differences between two studied groups of patients as regards liver function tests. As a traditional marker for HCC, AFP was significantly increased among HCC group of patients. Macroscopic examination of hepatocellular malignant lesions revealed that distribution of these lesions were more prevalent in both right and left lobes, then the right lobe and were least found in left lobe with mean size of 3.65 ± 1.75 cm. the frequency of single lesion was more than that of multiple lesions.

**Table 1:** Demographic; clinical; biochemical and pathological data of the two studied groups of patients

Variables		HCC group N= 20	Chronic hepatitis group N= 20
Age in year’s	mean± SD.	56.25 ± 8.13	46.22 ± 8.45

Sex			
Male No (%)		19 (95)	15(75)
Female: No (%)		1 (5)	5 (25)

PH of anti-Sch. ttt.			
Negative No (%)		9 (45)	8 (40)
Positive No (%)		11 (55)	12 (60)

AST IU/L	median (range).	98 (33-300)	53 (33-83)

ALT IU/L	median(range)	35.6 (16-221)	57 (28-192)

Albumin gm/dl	median(range)	2.5 (1.5-3.2)	2.8 (1.8-3.5)

Total bilirubin mg/dl	median(range)	1.6 (1-2.2)	1.4 (1-2.2)

AFP ng/ml	median (range).	21 (8-7460)	5 (1.30-75)

CHOL mg/dl	mean ± SD.	108.00 ± 16.35	121.66 ± 33.29

TRG mg/dl	mean ± SD.	91.20 ± 44.69	74.00 ± 13.11

HDL mg/dl	mean ± SD.	41.00 ± 18.29	28.00 ± 15.11

LDL mg/dl	mean ± SD.	42.30 ± 18.46	114.00 ± 16.35

HCC lesions Number:			
	1 No (%)	9 (45)	
	Multiple No (%)	11 (55)	
Site:			
	Rt. lobe No (%)	7 (35)	
	Lt. lobe No (%)	1 (5)	
	Both No (%)	12 (60)	
Size cm Grade:		3.65 ± 1.75	
	І No (%)	4 (20)	
	П No (%)	16 (80)	
Type:			
	Trabecular No (%)	8 (40)	
	Mixed No (%)	12 (60)	

Liver background:			
1-Mixed cirrhosis with mild activity No (%)		2 (10)	5 (25)
2-Mixed cirrhosis with moderate activity No (%)		18 (90)	15 (75)

PH of anti-Sch. ttt: past history of antischistosomal treatment. AST: aspartate transaminase; ALT: alanine transaminase; AFP: α-fetoprotein; CHOL: cholesterol; TRG: triglycerides; HDL: high density lipoprotein; LDL: low density lipoprotein.

### microRNAs expression

Calculated RQ expression of miRNAs 21;199a and 155 revealed that miRNAs 21 and 155 were over expressed in sera of patients with HCC compared to patients with chronic hepatitis (p < 0.0001) (Table 2). While serums mean values of miR 199a was significantly decreased among HCC group patients when compared to patients with chronic hepatitis (p < 0.0001) (Table 2). Correlation of serum miR 21 to age; tumor size and biochemical and molecular investigations revealed that miR 21 was directly correlated to α-fetoprotein (P = 0.006, significant); HDL (p < 0.0001, highly significant); miR -199 was inversely correlated to α-fetoprotein (P = 0.009, significant); miRNA 155 as directly correlated to α-fetoprotein (p = 0.001, significant) and serum total bilirubin (p = 0.037, significant). Otherwise, there was no significant correlation of serum miRNAs to other studied variables. There were no significant impacts of tumor characters as regards number; site; size and microscopic features on serum or tissue expression of both studied miRNAs.

**Table 2:** Comparison between HCC group and chronic hepatitis group as regards mean values of serum mirNAs and apoptotic markers

mirRNAs (Mean ± SD)	HCC group N = 20	Chronic hepatitis group N = 20	Controls N = 25	P value
Serum miR 21	7.76 ± 2.66	2.96 ± 1.92	1.2 ± 0.8	0.0001**
Serum miR 199-a	0.79 ± 0.62	2.15 ± 1.048	2.5 ± 1.01	0.0001**
Serum miR 155	10.25 ± 3.31	5.18 ± 2.45	1.1 ± 0.7	0.0001**
Serum PDCD4	12.78 ± 5.36	6.87 ± 1.76	4.37 ± 1.13	0.0001**
Serum CYTC	12.29 ± 4.87	7.63 ± 1.92	4.09 ± 0.92	0.01*

* : P value is significant;

** : P value is highly significant.

### Serum values of apoptotic markers

We found that serum levels of PCDC4 and CYTC were increased in patients with HCC when compared to chronic hepatitis patients. They were also increased in patients with chronic hepatitis when compared to controls (p < 0.05, significant) as shown in Table 2.

Correlations of apoptotic markers with clinical and biochemical investigations revealed that serum values of PDCD4 was directly correlated with age and α fetoprotein (p = 0.004; 0.029 respectively, significant). On the other hand, serum values of CYTC was inversely correlated with SGPT (p = 0.007, significant) and directly correlated with total serum bilirubin (p = 0.013, significant). Analyzing the impact of HCC characters on serum values of PDCD4 and CYTC proved that the mean values of both PDCD4 and CYTC were significantly higher in cases with single lesion of HCC (p < 0.05, significant). Right lobe location of HCC lesions has highest mean values of PDCD4 (p < 0.05, significant). As regards grade of differentiation, grade П have higher mean values of CYTC (p < 0.05, significant).

Correlations between apoptotic markers and miRNAs revealed that there was direct correlations between apoptotic markers and oncomirs miRNAs 21 and 155 while apoptotic markers were inversely correlated with miRNA 199-a as shown in Table 3.

**Table 3:** Correlations between serum mirNAs and apoptotic markers

Variables	Serum PDCD4	Serum CYTC
Serum miR 21 Mean ±SD	R = 0.538** P = 0.0001	R = 0 .570** P = 0.0001

Serum miR 199-a Mean ±SD	R = - 0.330* P = 0.041	R = - 0.511** P = 0.001

Serum miR 155 Mean ±SD	R =0.575** P = 0.0001	R = 0.334* P = 0.038

* : P value is significant;

** : P value is highly significant.

### Diagnostic accuracy of both microRNAs and apoptotic markers for HCC

As regards the diagnostic accuracy of the studied mirNAs and apoptotic markers for HCC, it was found that highest sensitivity was achieved by mir 21 and α fetoprotein while least sensitivity was achieved by CYTC. Serum α-fetoprotein has the least specificity while mir 21 and CYTC have the highest specificity. Combining mirNAs with each other resulted in 100% sensitivity and specificity except combined miR199-a and miR 155 have 88.90% specificity. Combined PDCD4 and CYTC can diagnose HCC with 94.70% sensitivity and 88.90% specificity as shown in Table 4.

**Table 4:** Diagnostic accuracy for both serum mirNAs and apoptotic markers as early detectors for HCC

Group	Parameter	Area under the curve	Cutoff value	Sensitivity %	Specificity %
HCC	Serum miR 21	0.981	3.800	100.0 %	88.90%

	Serum miR 199-a	0.843	1.150	83.30%	77.80%

	Serum miR 155	0.907	6.300	94.40%	77.80%

	Serum PDCD4	0.895	8.337	89.50%	77.80%

	Serum CYTC	0.798	9.610	63.20%	88.90%

	Serum α-fetoprotein	0.832	8.050	100.0 %	69.2 %

	combined miR 21 and miR 199-a	1.000		100.00%	100.00%

	combined miR 21 and miR 155	1.000		100.00%	100.00%

	combined miR199-a and miR 155	0.987		100.00%	88.90%

	Combined PDCD4 and CYTC	0.918		94.70%	88.90%

## Discussion

One hundred seventies (170) million people are infected with Hepatitis C virus (HCV) all over the world [13]. Progression of hepatic inflammation to liver fibrosis, advanced fibrosis and liver cirrhosis present the platform for HCC development [14]. It was supposed that contribution of the apoptotic process during liver fibro genesis may be important for chronicity persistence, failure of response to antiviral drugs, fibrosis progression, and liver carcinogenesis [15].

It was found that miRNAs represents the genetic signature for many diseases including liver cancer [16]. miRNAs usually target many genes, half of these genes are located in cancer-associated regions, including common chromosomal breakpoints, regions of loss of heterozygosity (LOH), amplified regions, fragile sites and hotspots for papilloma virus integration sites [17].

Apoptosis counterbalance the cell proliferation; so that, it is important for tissue homeostasis. Some of miRNAs influence cancer development might be by regulating apoptosis. This was approved by many studies [18].

Hou et al. used NGS, which is a high-throughput technology that supplies universal information on all miRNAs in only one sample [19]. They investigated the miRNomes in human normal liver, inflamed liver due to viral hepatitis, and liver cancer. They found nine miRNAs accounted for ~88.2% of the miRNome in human liver. The three most represented miRNAs were mRr-122, miR-192, and miR 199 a/b-3p. In HCC miR-199a/b-3p is lowered, this is accompanied with bad prognosis. Moreover, both in vitro and in vivo, the PAK4/Raf/MEK/ERK pathway is inhibited by miR-199a/b-3p targeting tumor-promoting PAK4 to suppress HCC growth.

We suggest that rate of hepatocyte death increases during liver carcinogenesis and this may explain high serum levels of PCDC4 and CYTC among patients with chronic hepatitis and HCC. All HCC cases in the current study have liver cirrhosis as a background. This background with continuous progression of inflammation may have an impact on increased serum values of apoptotic markers among cirrhotic and malignant patients.

Programmed cell death 4 (PDCD4) proteins is a translational repressor that blocks helicase activity leading to negative effects on/control of the inception of mRNA translation [20]. Some studies have found a task for PDCD4 as a tumor oppressor that is missed in definite aggressive malignancies [21]. Surprisingly, recent evidence also proved that the mission of PDCD4 can be changed by the cofactor protein arginine methyltransferase 5 (PRMT5) and that arginine methylation of PDCD4 leads to progression of malignant transformation [22]. Therefore, there is strong evidence that PDCD4 has a serious role in the regulation of carcinogenesis and that its deregulation has significant outcome in cell growth and carcinogenesis.

In our clinical practice, we find that late diagnosed HCC is an aggressive disease with high mortality rate; decreased survival rate and poor prognosis. So that search for novel molecular biomarkers for early detection of HCC will equal early therapeutic intervention; increased survival rate; better prognosis with improved quality of life for these patients.

In the current study, we research for the relation between serum values of microRNAs and serum values of apoptotic markers in a trial to provide an in-depth view of genetic alteration patterns occurring in HCC and enable the discovery the mechanism by which microRNAs act in pathogenesis of HCC that might potentially be new diagnostic and/or targets for HCC treatment

We found that apoptotic markers were directly correlated with oncomirs 21 and 155 while they were inversely correlated with tumor suppressor mir 199-a. Our findings were against findings of previous studies, as Chu and his colleagues who reported that oncomirs inhibit apoptosis and down regulated miRNAs induce apoptosis during pathogenesis of HCC [23].

Zhu and his colleagues demonstrated that miR-21 was increased in liver cancer tissues and cell lines and was a strong stimulator of the migrative/invasive abilities of malignant liver cells. They also proved that a miR-21 inhibitor restricted HepG2 cell immigration and metastasis by direct/indirect control of PDCD4 and downstream signaling pathway molecules (p-c-Jun/AP-1, MMP-2 and MMP-9), and that the transcription factor AP-1 directly activates miR-21 transcription. They, therefor, suggest that a positive feedback loop of miR-21-PDCD4-AP-1 maintains the miR-21-mediated biological effects of the liver cancer phenotype. They concluded that molecular introduction design interfering with the miR-21-PDCD4-AP-1 feedback loop might supply strong base for inhibiting invasion/metastasis in liver cancer in the near future [24].

Cheng and his colleagues used antisense RNA library to specifically knock down 90 human miRNAs in two different cell lines – HeLa (cervical carcinoma) and A549(lung carcinoma) – and tested for alterations in cell proliferation or apoptosis. In HeLa cells, inhibition of 19 miRNAs led to reduced cell growth, while inhibition of two miRNAs, miR-21 and miR-24, resulted in greater cell growth. Interestingly, inhibition of miR-24 in A549cell s led to the complete opposite phenotype: significant decreased of cell proliferation [25].

This result supposed that depending on the cellular environment or context, the same miRNA may have totally different effects. The ant proliferative activity of miR-21 in HeLa cells is also intriguing, because miR-21 has been proved to act as an ant apoptotic factor in human glioblastoma cells; therefore, one would have expected inhibition of miR-21 to result in a reduction of cell growth. Finally, inhibition of miR-21 in A549cells did not lead to either specific up- or downregulation of cell proliferation. Therefore, likely to miR-24, miR-21 might have different tasks in different organs. Cheng and his colleagues also reported that inhibition of seven miRNAs (miR-1d, 7, 148, 204, 210, 216 and 296) result in increased caspase-3 activity and one miRNA (miR-214) results in reduced activity. How the various miRNAs affect apoptosis remains to be investigated. Nevertheless, these experiments show the potential of such global screens to detect important candidate miRNAs, which can then be investigated further using more significant procedures. As discussed above, miR-21 can also act as an antiapoptotic factor. Glioblastoma, a highly malignant human brain cancer, strongly increased miR-21. Chan and his colleagues found greatly elevated levels of miR-21 in human glioblastoma malignat tissues, in early-passage glioblastoma cultures and in six established glioblastoma cell lines. Knock down of miR-21 in cultured glioblastoma cells led to a marked decrease in cell number. This reduction was not due to large differences in cell growth, but rather due to an increase in apoptosis, as detected by caspase-3 and -7 enzymatic activities and TdT-mediated dUTP nick-end labeling (TUNEL) staining. How miR-21 down regulates apoptosis, and whether it acts directly or indirectly remains to be detected [26].

Palma and his colleagues reported an anti-leukaemic role for miR-155 in human FLT3-wildtype AML, by initiating cell apoptosis and myelomonocytic differentiation, which is in contrast to its previously suggested role as an oncogene. This highlights the complexity of gene regulation by microRNAs that depending on disease context or tissue type may have tumor repressor or oncogenic effects [27]. Here in the current study we can report that deregulation of the studied microRNAs have significant roles in development of HCC. They are powerful early diagnostic noninvasive markers for HCC. HCC usually develops on cirrhosis in cases of HCV infection as we found that liver background among HCC patients was mixed cirrhosis and the activity was mostly moderate which support that the process of inflammation in patients with HCV related HCC is continuous. The underlying mechanism for liver carcinogenesis is completely complex.so that, elevated serum apoptotic markers among HCC patients may be referred to continuous inflammatory process of underlying cirrhosis. According to our findings, it seems that oncogenic microRNAs induce liver carcinogenesis in HCV patients irrespective of suppression of apoptosis and the down regulated microRNA did not induce apoptosis to control tumorigenesis among HCV patients. We can suggest another explanation for the relation between microRNAs and apoptotic markers in the present study which is the process of underlying inflammation during HCV infection is not only continuous but it also exceeds the process of carcinogenesis. However, our findings may pave the way for search for treatment that target apoptosis with treatment target overexpressed or replace down regulated microRNAs to control the underlying mediators of HCC pathogenesis.

In conclusion, both microRNAs and apoptotic markers have roles in HCC pathogenesis. They could be used as early diagnostic markers for HCC. Further efforts must be paid to pave the way for emerging therapies to target apoptotic markers and expressed micoRNAs to control HCC development.

## Author contribution

WME, KSA and MA proposed the study. RRK supplies tissues and blood samples. YAE, HHF and AEH help in collecting samples, all cooperate in writing the paper.
